# Determinants of circulating PCSK9 levels and the efficacy of PCSK9 inhibitor therapies in chronic kidney disease: a systematic review

**DOI:** 10.1007/s00228-025-03965-w

**Published:** 2026-01-17

**Authors:** Tatjana Ábel, Béla Benczúr, Éva Csobod Csajbókné

**Affiliations:** 1 Department of Dietetics and Nutritional Sciences, Faculty of Health Sciences, Semmelweis University, Budapest, Hungary; 2 Dept. of Internal Medicine (Cardiology/Nephrology), János Balassa County Hospital, Szekszárd, Hungary

**Keywords:** Alirocumab, Atherosclerotic cardiovascular disease, Chronic kidney disease, Evolocumab, Inclisiran, Low-density lipoprotein cholesterol, PCSK9

## Abstract

**Purpose of review:**

Chronic kidney disease (CKD) significantly increases the risk of atherosclerotic cardiovascular disease (ASCVD), with dyslipidemia—particularly elevated low-density lipoprotein cholesterol (LDL-C)—being a shared risk factor. While statin-based therapies are effective in early-stage CKD, their benefits in dialysis and kidney transplant patients remain inconclusive. Proprotein convertase subtilisin/kexin type 9 (PCSK9) is a key regulator of lipid metabolism. This review aims to summarize the factors influencing PCSK9 levels in CKD patients and evaluate the safety and efficacy of PCSK9 inhibitors across different stages of CKD.

**Recent findings:**

Multiple factors have been associated with variations in plasma PCSK9 concentrations in CKD, including glycemic status, proteinuria, renal function, dialysis modality, lipid-lowering therapy, and circadian rhythms. PCSK9 inhibitors, such as alirocumab, evolocumab, and inclisiran, effectively reduce LDL-C and ASCVD risk in patients with mild-to-moderate CKD. However, evidence is limited in patients with advanced CKD (stages 4–5, end stage renal disease (ERSD)), particularly those undergoing dialysis.

**Summary:**

Elevated PCSK9 levels may not independently predict ASCVD risk in CKD populations. Nonetheless, PCSK9 inhibitors provide a well-tolerated and effective lipid-lowering option in early CKD. Large-scale prospective studies are warranted to clarify their role in patients with ESRD.

**Supplementary Information:**

The online version contains supplementary material available at 10.1007/s00228-025-03965-w.

## Introduction

Atherosclerotic cardiovascular disease (ASCVD) remains a leading cause of morbidity and mortality worldwide [1, 2]. Elevated levels of low-density lipoprotein cholesterol (LDL-C) are not only a modifiable risk factor but a principal contributor to ASCVD pathogenesis [3, 4]. Statins constitute the cornerstone of LDL-C reduction, typically achieving a 30–60% decrease [3, 4]. However, a considerable proportion of patients fail to reach guideline-recommended LDL-C targets or experience statin intolerance [3–7]. This shortfall is further exacerbated by progressively lower LDL-C goals for high- and very high-risk patients, which are often unattainable with statin monotherapy, even at maximal tolerated doses. While ezetimibe provides additional LDL-C lowering of 18–25%, its impact is modest when used alone [8].

Chronic kidney disease (CKD) is defined as persistent abnormalities in kidney structure or function for more than three months, regardless of etiology [9]. Affecting over 800 million individuals globally, CKD is associated with disproportionately high ASCVD burden [9–11]. According to current European cardiovascular prevention guidelines, patients with CKD are stratified into high or very high-risk categories, necessitating rigorous LDL-C targets [11]. While statins remain effective for primary and secondary cardiovascular prevention in early-stage CKD, landmark trials (4D, AURORA, SHARP) have demonstrated limited or equivocal benefits in patients on dialysis or post-transplant [12–18].

The 2024 KDIGO guidelines recommend statin monotherapy or statin-ezetimibe combination for dyslipidemia management in CKD but refrain from routine statin use in dialysis-dependent individuals [9]. Notably, proprotein convertase subtilisin/kexin type 9 (PCSK9) inhibitors are endorsed as adjuncts to maximally tolerated statin therapy for patients with CKD and familial hypercholesterolemia or clinical ASCVD who do not achieve LDL-C targets [9].

Statins, while lowering LDL-C, paradoxically upregulate circulating PCSK9 levels—especially in individuals with ASCVD—potentially diminishing therapeutic efficacy [19]. PCSK9 inhibitors represent a novel class of lipid-lowering agents [20]. Alirocumab and evolocumab, monoclonal antibodies, enhance LDL receptor (LDLR) recycling and LDL-C clearance, whereas inclisiran, a small interfering RNA (siRNA), suppresses hepatic PCSK9 synthesis [21, 22]. All three agents have demonstrated favorable efficacy and safety profiles [22, 23].

This systematic review aims to (1) elucidate determinants of PCSK9 levels in CKD and (2) evaluate the clinical utility of PCSK9 inhibitors across various stages of CKD.

## Methods

**Fig. 1:**
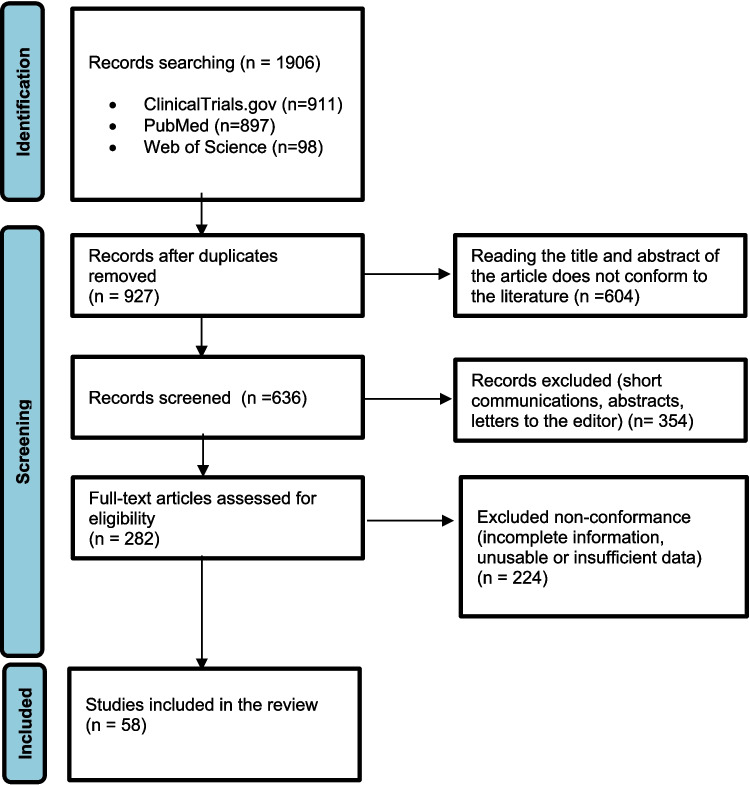
Flow chart showing inclusion and exclusion of literature

A comprehensive literature search was conducted using the electronic databases PubMed, Web of Science, and ClinicalTrials.gov. The following keywords were used: “evolocumab”, “alirocumab”, “inclisiran”, “PCSK9 inhibitor”, “PCSK9”, “chronic kidney disease”, and “CKD”. The search aimed to identify original studies, systematic reviews, and relevant clinical trials examining PCSK9 levels and PCSK9 inhibitor therapies in patients with CKD across different stages (Fig. 1). Our systematic review complies with the statement of Preferred Reporting Items for Systematic Reviews [24]. In addition to the systematic review methods described, we conducted a methodological quality appraisal of all cited meta-analyses using the AMSTAR 2 tool (16-item checklist). This appraisal assessed protocol registration, literature search, duplicate study selection and data extraction, provision of excluded studies, risk of bias assessment, publication bias assessment, and other key domains. For the purposes of this review, the term “advanced CKD” refers to CKD stages 4–5, including patients with end-stage renal disease (ESRD).

### Dyslipidemia in chronic kidney disease

Patients with CKD represent a heterogeneous population characterized by diverse etiologies, varying degrees of glomerular filtration rate (GFR) impairment (G1–G5), degrees of proteinuria (A1–A3), and coexisting conditions such as diabetes and hypertension [9, 25, 26]. Additionally, the type and modality of renal replacement therapy may further influence lipid metabolism.

CKD is associated with significant alterations in lipid metabolism, often manifesting as elevated triglycerides, reduced high-density lipoprotein cholesterol (HDL-C), and variable levels of total and LDL cholesterol [12, 27–33]. In the absence of albuminuria, declining GFR is typically correlated with reductions in both LDL-C and HDL-C [27]. However, in patients with albuminuria, total cholesterol and LDL-C levels tend to rise in conjunction with lower HDL-C [34].

Hypertriglyceridemia in CKD may arise from impaired catabolism of triglyceride-rich lipoproteins due to decreased activity of lecithin: cholesterol acyltransferase (LCAT), reduced expression of very low-density lipoprotein (VLDL) receptors and lipoprotein lipase (LPL), and acquired LDL receptor (LDLR) deficiency seen in proteinuric states [35–37].

HDL metabolism is also disrupted in CKD, contributing to diminished anti-inflammatory and antioxidant functions [38–41]. Reduced hepatic synthesis of apolipoprotein A-I (ApoA-I), decreased LCAT activity, and impaired cholesterol efflux—due to downregulation of ATP-binding cassette transporters (ABCA1, ABCG1)—are key contributors [42–44]. Increased activation of cholesterol ester transfer protein (CETP) in CKD further lowers HDL-C levels [45].

Despite only modest elevations in LDL-C, qualitative changes in LDL particles, including a higher proportion of small dense LDL, are more atherogenic [46, 47]. These shifts are attributed to increased cholesterol acyltransferase (ACAT) activity and reduced LDLR expression in the proteinuric stage of CKD [37, 40].

Lipoprotein(a) [Lp(a)] comprises an LDL-like particle with apolipoprotein(a) covalently bound to apolipoprotein B-100 [48]. Lp(a) levels are largely genetically determined and associated with increased risk of foam cell formation, inflammation, oxidative stress, and thrombosis [49, 50]. Recent guidelines recognize Lp(a) as a therapeutic target for residual ASCVD risk [51–54]. Elevated Lp(a) levels are independently associated with GFR decline and may appear early in CKD, rising dramatically in nephrotic syndrome [52–56].

### Factors affecting pcsk9 levels in ckd

Studies investigating PCSK9 levels in CKD have yielded inconsistent results [57–61]. In two cohort studies (CARE FOR HOMe and LURIC), Rogacev et al. reported no association between PCSK9 concentrations and GFR in patients with stage G2–G4 CKD [57]. Similarly, Morena et al. and Kheirkhah et al. found no significant correlations between PCSK9 levels and GFR or proteinuria in non-dialysis CKD patients [58, 59]. Conversely, Konarzewski et al. observed higher PCSK9 levels in CKD patients and demonstrated an inverse correlation with GFR, including individuals on hemodialysis or post-transplant [60]. Abujrad et al. reported lower PCSK9 levels in hemodialysis patients compared to non-CKD controls [61].

Such discrepancies likely stem from differences in study design, patient selection, and clinical heterogeneity. PCSK9 levels may be modulated by a variety of physiological and pathological factors (Table 1).

**Table 1:** Potential factors affecting PCSK9 levels in patients with CKD

Factors	References
Fasting glucose, insulin, HOMA-IR*, HbA1c**	Ruscica et al. [62]; Macchi et al. [63]
Circadian rhythm, fasting state	Abujrad et al. [61]
Proteinuria	Jin et al. [64]; Dorion et al. [65]
ESRD*** (treated with peritoneal dialysis, or hemodialysis)	Jin et al. [64]; Rasmussen et al. [66]
Lipid-lowering treatment (statin, ezetimibe, statin/fibrate combination)	Rogacev et al. [57]; Dubuc et al. [67]; Elewa et al. [68]
ASCVD****	Kheirkhah et al. [59]; Vlad et al. [69]; Strålberg et al. [70]; Kajingulu et al. [71]

This table lists factors influencing PCSK9 levels (e.g., metabolic, renal, and treatment-related parameters) together with relevant literature references.

* HOMA-IR = homeostasis model assessment of insulin resistance; ** HbA1c = Hemoglobin A1c; *** ESRD = end-stage renal disease; **** ASCVD = atherosclerotic cardiovascular disease.

### Glycemic control and insulin resistance

Several studies have shown that PCSK9 levels positively correlate with fasting glucose, insulin, homeostasis model assessment of insulin resistance (HOMA-IR), and hemoglobin A1c (HbA1c) [62, 63]. In contrast, Da Dalt et al. suggested that PCSK9 deficiency may reduce insulin secretion, contributing to hyperglycemia [72]. Interestingly, in Abujrad’s study, despite nearly half of CKD patients being diabetic, PCSK9 levels were lower, suggesting that factors beyond glycemic status influence PCSK9 in CKD [61].

### Circadian rhythm and fasting effects

PCSK9 exhibits a circadian rhythm similar to cholesterol synthesis, with peak levels occurring at night [73]. Statins and bile acid sequestrants can blunt this rhythm [73]. Fasting significantly reduces circulating PCSK9 levels, with reported reductions of up to 35% following prolonged fasting [73]. Timing of blood sampling in relation to meals and dialysis sessions may therefore influence measured PCSK9 levels, complicating intergroup comparisons [61, 73, 74].

### Proteinuria

Proteinuric CKD is associated with elevated LDL-C and Lp(a) levels, potentially driven by increased PCSK9 levels [75, 76]. PCSK9 promotes LDLR degradation, impairing LDL clearance [64, 77, 78]. Increased hepatic and renal PCSK9 expression and reduced clearance may contribute to hypercholesterolemia in nephrotic syndrome [79, 80]. Molina-Jijon et al. demonstrated that renal PCSK9 silencing in mice ameliorated nephrotic dyslipidemia [80]. Multiple studies have confirmed strong correlations between PCSK9 and LDL-C in proteinuric states [64, 77, 65, 81, 82].

### End-Stage Renal Disease (ESRD) and dialysis

Lipid profiles vary by dialysis method [64, 83]. Hemodialysis patients often have normal cholesterol levels, whereas peritoneal dialysis patients typically exhibit marked hyperlipidemia. Jin et al. and Rasmussen et al. reported significantly higher PCSK9 levels in peritoneal dialysis patients [64, 66]. Protein losses in dialysate may stimulate PCSK9 expression [64]. In contrast, PCSK9 levels in hemodialysis patients were lower in studies by Konarzewski and Abujrad, possibly reflecting PCSK9 clearance during dialysis or suppression by uremic factors [60, 61].

### Lipid-lowering therapies

Statin therapy increases circulating PCSK9 levels [19, 84–86]. High-intensity statin treatment was associated with a significant increase PCSK9 levels [84–86]. In their meta-analysis, Sahebkar et al. found that lipophilic statins (atorvastatin, simvastatin, and pitavastatin) increased circulating PCSK9 levels to a greater extent than hydrophilic statins (rosuvastatin, pravastatin) [87].

Atorvastatin and rosuvastatin increased PCSK9 concentrations by 34–45% within a few weeks in a dose-dependent manner [67, 85]. The exact reason is unknown, but statins reduce intracellular cholesterol levels in the liver, which in turn activates the sterol regulatory element-binding protein 2 (SREBP2) pathway, increasing LDLR and PCSK9 mRNA expression [88–90].

As a result, most studies published in recent years have evaluated CKD patients taking statins separately from those not taking statins. However, the results are contradictory. Similar to non-CKD patients, Rogacev et al. also found higher PCSK9 concentrations in statin-treated CKD patients than in patients not taking statins [57]. According to the results of Abudraj et al., serum PCSK9 levels in hemodialyzed CKD patients did not differ significantly from those in a control group without CKD [61]. Furthermore, contrary to the results in hemodialysis patients, no association was found between PCSK9 concentration and total cholesterol and LDL-C levels in hemodialysis patients taking statins [61]. Hwang et al. obtained the same result in hemodialysis patients with CKD, i.e., no association was found between PCSK9 levels and lipid fractions [91].

Results to date show that ezetimibe treatment increases circulating PCSK9 levels, although this increase was no longer significant when ezetimibe was combined with statin therapy [67]. The lower LDL-C-lowering effect of ezetimibe may explain this phenomenon [67].

According to the results of a recently published study, fibrates may have a beneficial effect on reducing cardiovascular events in patients with moderate CKD and hypertriglyceridemia but LDL-C < 100 mg/dl who are not taking other lipid-lowering agents [92]. However, a meta-analysis by Sahebkar et al. showed that fibrate therapy significantly increases PCSK9 concentrations, which may partly explain why fibrates have little effect on LDL-C levels [93]. Elewa et al. studied diabetic patients with CKD (GFR categories: G1-G4, albuminuria categories A1-A3) and found that among lipid-lowering treatments, the combination of fibrates and statins was primarily associated with an increase in PCSK9 levels [68]. Fibrates reduce triglyceride levels and increase HDL-C levels by stimulating peroxisome proliferator-activated receptor alpha (PPAR-alpha) [94]. Their effect enhances fatty acid oxidation and lipoprotein lipase activity, as well as cholesterol excretion from the liver [95]. These effects may indirectly reduce intracellular cholesterol levels in the liver and thus secondarily increase PCSK9 expression and secretion [84, 96].

### PCSK9 levels and risk of ASCVD

Elevated circulating PCSK9 levels have been associated with several cardiometabolic risk factors, including high LDL-C, hypertriglyceridemia, hypertension, and type 2 diabetes [97]. Shi et al. demonstrated a significant association between PCSK9 and cardiovascular risk in women, while in men the correlation was limited to LDL-C [97]. Qiu et al. reported a 25% increased incidence of cardiovascular events among individuals in the highest PCSK9 quartile, with a nonlinear dose–response pattern [98]. Meta-analyses by Zhou et al. and Liu et al. confirmed that high PCSK9 levels are associated with a significantly increased risk of major adverse cardiovascular events (MACE), although the associations with stroke and all-cause mortality were inconsistent [99, 100].

In CKD populations, evidence remains limited and mixed [101–105]. Kheirkhah et al. and Vlad et al. found significant associations between elevated PCSK9 and ASCVD risk, independent of traditional risk factors [59, 69]. However, Rogacev et al. did not observe such associations [57]. Rasmussen et al. found no predictive value of PCSK9 in patients awaiting kidney transplantation [66]. Strålberg et al. identified a U-shaped relationship between PCSK9 and all-cause mortality in hemodialysis patients, while Kajingulu et al. reported a strong link between plasma PCSK9 and incident ASCVD in Black African patients on dialysis [70, 71]. These discrepancies likely reflect differences in patient characteristics, study design, and sample size (Table 2).

**Table 2:** PCSK9 levels and ASCVD in patients with CKD

Main finding on PCSK9 level and ASCVD link	Patients/CKD stage	References
PCSK9 independently predicts ASCVD*	Patients with Stage 3 CKD	Kheirkhah et al. [59]
Elevated PCSK9 linked to ASCVD* events	Mixed CKD stages, patients with dyslipidemia	Vald et al. [69]
Strong association with ASCVD* and all-cause mortality	Patients with ESRD** (treated with hemodialysis), black African	Kajingulu et al. [71]
No predictive value for cardiovascular events	Patients with Stage 2–4 CKD	Rogacev et al. [57]
No association with outcomes	Patients with ESRD** awaiting transplantation	Rasmussen et al. [66]
U-shaped relationship with ASCVD* mortality	Patients with ESRD** (treated with hemodialysis)	StrÃ¥lberg et al. [70]

This table summarizes the key findings on the association between PCSK9 levels and ASCVD* in patients with CKD at various stages, highlighting differences in predictive value and associations with cardiovascular outcomes or mortality.

*ASCVD = atherosclerotic cardiovascular disease; **ESRD = end-stage renal disease;

## PCSK9 inhibitor therapy in patients with CKD

Limitations in the efficacy and tolerability of statin therapy in patients with CKD, especially ESRD, necessitate the development of new effective lipid-lowering agents. Alirocumab and evolocumab, two monoclonal antibodies, and inclisiran, a long-acting synthetic siRNA targeting PCSK9, are approved for clinical use as lipid-lowering treatment. PCSK9 inhibitors appear to be well tolerated and effective in lipid lowering [106–110].

The results so far show that no changes in renal function were found during the treatment of CKD patients with alirocumab or evolocumab (Table 3) [111, 120]. In contrast to the other two PCSK9 inhibitors, inclisiran has been marketed more recently and little data has been published on its effects in CKD patients.

**Table 3:** Efficacy and safety of PCSK9 inhibitor therapy in patients with CKD

Efficacy and safety	Patients	PCSK9 inhibitors Intervention	References
Not increase the rate of new-onset diabetes, GFR is substantially unchanged	15.034 with stage 2 CKD and 4.443 with ≥ stage 3 CKD	Evolocumab	Charytan et al. [111]
Not change proteinuria	20 primary nephrotic syndrome, stage 0–3 CKD,	Alirocumab or evolocumab	Jatem et al. [112]
Significantly decreased proteinuria, GFR unchanged	76 with CKD, stage 1 to 4 CKD	PCSK9 inbibitors	Ramos et al. [113]
	68 yrs**, hemodialysis 61 yrs and 65 yrs hemodialysis 33 yrs, peritoneal dialysis 79 yrs, kidney transplant	Alirocumab Evolocumab Evolocumab Inclisiran	Dousdampanis et al. [114] Ishii et al. [115] Sanchidrián et al. [116] Ueberdiek et al. [117]
Effective in improving the lipid profile, reducing the risk of ASCVD*** (mainly for CKD < 3 CKD stage)	316 (eGFR 30–60 ml/min/1.73m^2^) 720 (eGFR 30–60 ml/min/1.73m^2^) 467 (eGFR 30–59 ml/min/1.73m^2^) 15.034 with stage 2 CKD and 4.443 with ≥ stage 3 CKD	Alirocumab Alirocumab Alirocumab Evolocumab	Kereiakes et al. [118] Cannon et al. [119] Toth et al. [120] Charytan et al. [111]

This table presents evidence on the efficacy and safety of PCSK9 inhibitors in patients with CKD, showing their effects on the incidence of diabetes, proteinuria, lipid profile, and LDL cholesterol targets. The studies cover different stages of CKD and treatment settings, including dialysis and transplantation.

* ESRD: End-stage renal disease.

** yrs: years.

*** ASCVD: Atherosclerotic cardiovascular disease.

### Glycemic effects

Most evidence suggests PCSK9 inhibitors do not increase the risk of new-onset diabetes [121]. A network meta-analysis by Pamporis et al. found no significant changes in glycemic parameters [122]. In the meta-analysis, Ma et al. observed an increase in the incidence of new diabetes in association with the use of PCSK9 inhibitors and high-intensity statin combination therapy [123]. However, it is important to note that when PCSK9 inhibitors and high-intensity statin combination therapy were compared with high-intensity statin monotherapy, the addition of PCSK9 inhibitors did not further increase the incidence of new diabetes. The FOURIER trial subgroup analysis including over 11, 000 diabetic and prediabetic patients showed no difference in fasting glucose or HbA1c between evolocumab and placebo [124]. A slight numerical increase in diabetes incidence in patients with CKD stage > 3 was not statistically significant [111].

### Proteinuria

Limited data suggest PCSK9 inhibitors may not adversely affect proteinuria [112, 113]. Jatem et al. found no significant change in proteinuria or serum albumin in patients with refractory nephrotic syndrome treated with alirocumab or evolocumab [112]. Ramos et al. reported a significant decrease in proteinuria (from 57 mg/g to 30 mg/g) after one year of PCSK9 inhibitor treatment, with preserved renal function [113].

### Efficacy and safety in patients with ESRD

To date, only case studies have been published on the use of PCSK9 inhibitors in patients undergoing hemodialysis, peritoneal dialysis, and kidney transplantation [114–117].

Dousdampanis et al. reported a case of a 68-year-old male patient with type 2 diabetes mellitus, ESRD treated with hemodialysis and hypercholesterolemia [114]. The patient had a history of ASCVD and was receiving treatment with a combination of statins and ezetimibe, but also had chronic hepatitis C infection and liver damage. Due to the potential hepatotoxic effect, the authors decided to discontinue the combined cholesterol-lowering therapy and instead administered 150 mg of alirocumab every two weeks for eight weeks for observation. As a result of alirocumab treatment, the patient’s LDL-C level decreased to 48 mg/dl, and the patient did not report any side effects [114].

The study by Ishii et al. described two brothers [115]. The 61- and 65-year-old men had a family history of hypercholesterolemia, ASCVD positivity, type 2 diabetes, and diabetic nephropathy. Both suffered from ESRD and were undergoing hemodialysis and lipoprotein apheresis treatment. Both men received combined cholesterol-lowering therapy with rosuvastatin (at various doses) and ezetimibe, but did not achieve their LDL-C target values. Therefore, the authors started them on evolocumab at a dose of 140 mg every two weeks, followed by 420 mg once a month. The younger men’s LDL-C levels decreased from 140 to 34 mg/dL, and the older men’s from 110 to below 50 mg/dL. A few years after starting evolocumab treatment, lipoprotein apheresis could be discontinued in both patients, as there were no signs of ASCVD progression or recurrence. The patients did not experience any side effects after starting evolocumab treatment [115].

Sanchidrián et al. reported the case of a 33-year-old man with ASCVD, heterozygous familial hypercholesterolemia, and ESRD treated with peritoneal dialysis [116]. The authors prescribed evolocumab (140 mg every two weeks) in addition to rosuvastatin and ezetimibe because the patient’s LDL-C level did not reach the target value. As a result of evolocumab treatment, the patient’s LDL-C level decreased to 50 mg/dl, and no side effects were observed during follow-up [116].

Ueberdiek et al. presented the case of a 79-year-old man who had one kidney removed due to carcinoma and nephrosclerosis in the other kidney [117]. Therefore, he received a kidney transplant from a deceased donor 12 years before starting inclisiran treatment. The patient had ASCVD. Despite treatment with atorvastatin and ezetimibe, LDL-C did not reach the target level, so treatment with 284 mg of inclisiran was started at 0, then 3 months, and then every 6 months. No side effects were reported during 1 year of inclisiran treatment, and LDL-C levels decreased significantly [117].

### Effectiveness on lipid profile and risk of ASCVD

Two recently published meta-analyses compared the efficacy of evolocumab, alirocumab, and inclisiran in terms of ASCVD and LDL-C, but did not report data on renal function [122, 125]. In a network meta-analysis by Pamporis et al., all three PCSK9 inhibitors significantly reduced MACE compared with the control group (inclisiran (RR = 0. 76, 95% CI [0. 61, 0.94], p-score = 0. 82), evolocumab (RR = 0. 78, 95% CI [0. 72, 0.86], p = 0. 76) and alirocumab (RR = 0. 85, 95% CI [0. 79, 0. 92], *p =* 0. 42) [122]. Alirocumab and evolocumab reduce the rate of cerebrovascular events, coronary revascularizations, and myocardial infarctions. Alirocumab treatment reduces all-cause mortality, while evolocumab is associated with a reduction in cardiovascular mortality.

In a meta-analysis, Imran et al. demonstrated that evolocumab reduced LDL-C levels by 61. 09%, alirocumab by 46. 35%, and inclisiran (284 mg) by 54. 83% [125]. Both evolocumab and alirocumab reduced the incidence of myocardial infarction and overall MACE. In addition, evolocumab reduced the incidence of coronary revascularization and stroke. Alirocumab treatment was associated with a reduction in cardiovascular mortality and all-cause mortality [125].

Patients with CKD (eGFR 30–60 ml/min/1. 73 m^2^) were also included in the previously published ODYSSEY COMBO I and ODYSSEY COMBO II studies [118, 119]. In these studies, the primary endpoint was the change in LDL-C levels in patients at high cardiovascular risk. These patients were receiving maximally tolerated statin therapy and had (i) LDL-C ≥ 70 mg/dl and established ASCVD, or (ii) LDL-C ≥ 100 mg/dl and a condition associated with coronary heart disease risk (e.g., CKD). A subgroup analysis of the COMBO I study showed that alirocumab treatment reduced LDL-C levels by 48% in patients with CKD (n = 36) and by a similar 50% in patients without CKD [125]. Neither ODYSSEY COMBO II nor the two-year follow-up study found a significant difference in the effect of alirocumab treatment on LDL-C between patients with and without CKD [119, 126].

The largest study published to date is the clinical trial by Toth et al., which summarized the results of eight randomized, placebo-controlled, double-blind ODYSSEY phase studies [120]. The study included 4629 patients with hypercholesterolemia (99% treated with statins), without renal impairment (GFR ≥ 60 ml/min/1. 73 m^2^) or with renal impairment (n = 467; GFR 30–59 ml/min/1. 73 m^2^) who received alirocumab compared with placebo or ezetimibe [120]. LDL-C levels in patients treated with alirocumab decreased to a similar extent compared with baseline at week 24 of the study, regardless of renal function impairment (46.1–62. 2%) or absence of impairment (48.3–60. 1%) [120]. Similar changes were observed in lipoprotein (a), low-density lipoprotein cholesterol, apolipoprotein B, and triglyceride levels [120].

A total of 27, 565 patients treated with statins participated in the Fourier study, who were randomized in a 1: 1 ratio to receive evolocumab (140 mg every two weeks or 420 mg monthly) or placebo [111]. Of the patients enrolled, 15, 034 had stage 2 CKD and 4, 434 had stage ≥ 3 CKD. A 59% reduction in LDL-C was observed in the evolocumab-treated group, regardless of whether patients had CKD or not [111]. The primary endpoints of the study were cardiovascular death, myocardial infarction, stroke, hospitalization for unstable angina, or coronary revascularization. The most important secondary endpoints were cardiovascular death, myocardial infarction, or stroke. The relative risk reduction was similar for both primary and key secondary endpoints in all patients with CKD. The absolute reduction in the composite endpoint of cardiovascular mortality, MI, or stroke was numerically greater with evolocumab treatment in patients with more advanced CKD (stage 4–5, including ESRD) [111].

## Discussion

Circulating PCSK9 levels in CKD are influenced by a complex interplay of metabolic, renal, and treatment-related factors. This heterogeneity may explain the inconsistent findings across studies regarding the association of PCSK9 with GFR, proteinuria, and dialysis method. In patients on dialysis, interpreting PCSK9 levels is particularly challenging due to variability in blood sampling protocols, feeding status, and clearance mechanisms.

The predictive value of PCSK9 for ASCVD in CKD populations is less well established than in the general population. While several cohort studies support a link between elevated PCSK9 and ASCVD risk, findings remain inconsistent, likely due to differences in study design, patient characteristics, CKD stages, and event rates.

Statin therapy, though foundational in ASCVD prevention, induces PCSK9 expression, potentially attenuating its LDL-C lowering efficacy. This phenomenon is particularly relevant in CKD patients, where statin intolerance or resistance is common, and residual cardiovascular risk remains high. In contrast, PCSK9 inhibitors lower LDL-C independently of renal function and without the compensatory PCSK9 upregulation seen with statins. Although PCSK9 expression has been identified in renal tissues, the clinical implications of this remain unclear [127, 128].

Alirocumab and evolocumab have demonstrated consistent LDL-C reduction and ASCVD risk lowering in patients with mild-to-moderate CKD, without significant effects on renal function [129]. Evidence regarding inclisiran in CKD is limited but promising. Importantly, PCSK9 inhibitors do not appear to increase the risk of new-onset diabetes or worsen proteinuria.

Statin therapy has limited benefit in dialysis and transplant patients, a population at particularly high risk for ASCVD. Case reports suggest that PCSK9 inhibitors may be effective in these groups, but robust randomized trials are needed to confirm their safety and efficacy in end-stage renal disease.

In this review, we used the AMSTAR 2 tool to assess the methodological quality of all explicitly referenced meta-analyses, which allows for a comprehensive assessment of the rigor of systematic reviews. Two meta-analyses demonstrated high methodological quality, supported by prior protocol registration, comprehensive literature search, and reliable assessment of biases [100, 122]. Three analyses received a moderate rating, primarily due to the lack of protocol registration and/or failure to provide a detailed list of excluded studies [22, 99, 125]. The earliest meta-analysis cited was also rated as moderate, reflecting less rigorous reporting practices at the time of publication [87]. These methodological quality profiles are important for interpreting the reliability of the pooled estimates and should be taken into account when drawing conclusions from the available evidence.

Given the heterogeneity of studies of PCSK9 inhibitors in CKD populations, a specific meta-analysis summarizing data from all available randomized controlled trials and high-quality observational studies would be extremely valuable. Such an analysis could clarify the effect of treatment on cardiovascular and renal outcomes in different stages of CKD. Incorporating this approach into future research would help to clarify the scope and applicability of current findings.

In addition, future research should focus on several practical areas, such as long-term, randomized trials with PCSK9 inhibitors specifically in advanced stages of CKD, investigation of their effects on kidney function, such as GFR decline and proteinuria, assessing cost-effectiveness in CKD populations, and investigating potential interactions with new lipid-lowering therapies. Multiethnic and multicenter collaborations will also be essential to ensure the generalizability of findings.

This review has limitations. The studies included varied in the number of patients, age, race, and CKD stage, and included patients in different cardiovascular risk groups. All of these factors made comparisons difficult and led to some differences in results.

## Conclusion

Current evidence suggests that PCSK9 levels in CKD are modulated by diverse metabolic and renal factors, complicating their interpretation as biomarkers. PCSK9 inhibitors, particularly alirocumab and evolocumab, provide safe and effective LDL-C lowering in patients with mild-to-moderate CKD and may reduce ASCVD risk. Their role in advanced CKD and ESRD is less well defined, though preliminary data are encouraging. Well-designed, prospective studies are required to clarify their impact on cardiovascular outcomes in patients with end-stage kidney disease.

## Supplementary Information

Below is the link to the electronic supplementary material.

Supplementary file1 (PPTX 52 KB)

Supplementary file2 (PPTX 57 KB)

## Data Availability

No datasets were generated or analysed during the current study.

## Abbreviations

ABC: ATP-binding cassette
ACAT: Cholesterol acyltransferase
ApoA-I: Apolipoprotein A-I
ASCVD: Atherosclerotic cardiovascular disease
CETP: Cholesterol ester transfer protein
CKD: Chronic kidney disease
ESRD: End-stage renal disease
GFR: Glomerular filtration rate
HbA1c: Hemoglobin A1c
HDL-C: High-density lipoprotein cholesterol
HOMA-IR: Homeostasis model assessment of insulin resistance
IDL: Intermediate density lipoprotein
KDIGO: Kidney Disease Improving Global Outcomes
LCAT: Lecithin: cholesterol acyltransferase
LDL-C: Low-density lipoprotein cholesterol
LDLR: LDL-receptor
Lp(a): Lipoprotein (a)
LPL: Lipoprotein lipase
MACE: Major adverse cardiovascular event
MI: Myocardial infarction
PCSK9: Proprotein convertase subtilisin/kexin type 9
siRNA: Small interfering RNA
SREBP2: Sterol regulatory element-binding protein-2 (SREBP2)?
UACR: Urine albumin-creatinine ratio
VLDL-C: Very low-density lipoprotein cholesterol
