# Supplementary material for: Determinants of circulating PCSK9 levels and the efficacy of PCSK9 inhibitor therapies in chronic kidney disease: a systematic review
Source: Eur J Clin Pharmacol. 2026 Jan 17;82(2):37. doi: 10.1007/s00228-025-03965-w (PMC12812102; doi:10.1007/s00228-025-03965-w)
Supplement: Supplementary file 1 — Supplementary file1 (PPTX 52 KB) [file 228_2025_3965_MOESM1_ESM.pptx]

## Slide 1
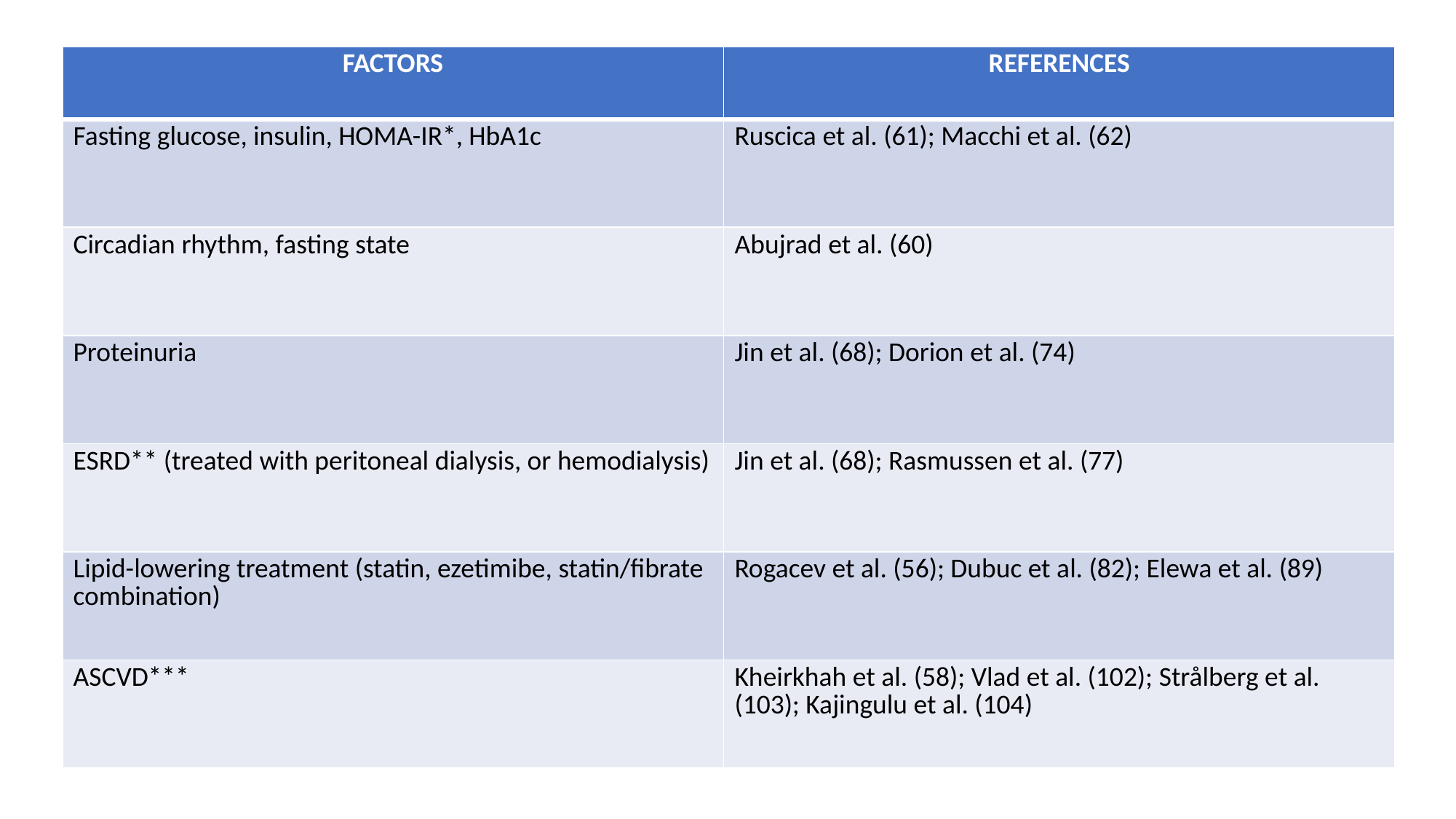

| FACTORS | REFERENCES |
| --- | --- |
| Fasting glucose, insulin, HOMA-IR\*, HbA1c | Ruscica et al. (61); Macchi et al. (62) |
| Circadian rhythm, fasting state | Abujrad et al. (60) |
| Proteinuria | Jin et al. (68); Dorion et al. (74) |
| ESRD\*\* (treated with peritoneal dialysis, or hemodialysis) | Jin et al. (68); Rasmussen et al. (77) |
| Lipid-lowering treatment (statin, ezetimibe, statin/fibrate combination) | Rogacev et al. (56); Dubuc et al. (82); Elewa et al. (89) |
| ASCVD\*\*\* | Kheirkhah et al. (58); Vlad et al. (102); Strålberg et al. (103); Kajingulu et al. (104) |

## Slide 2
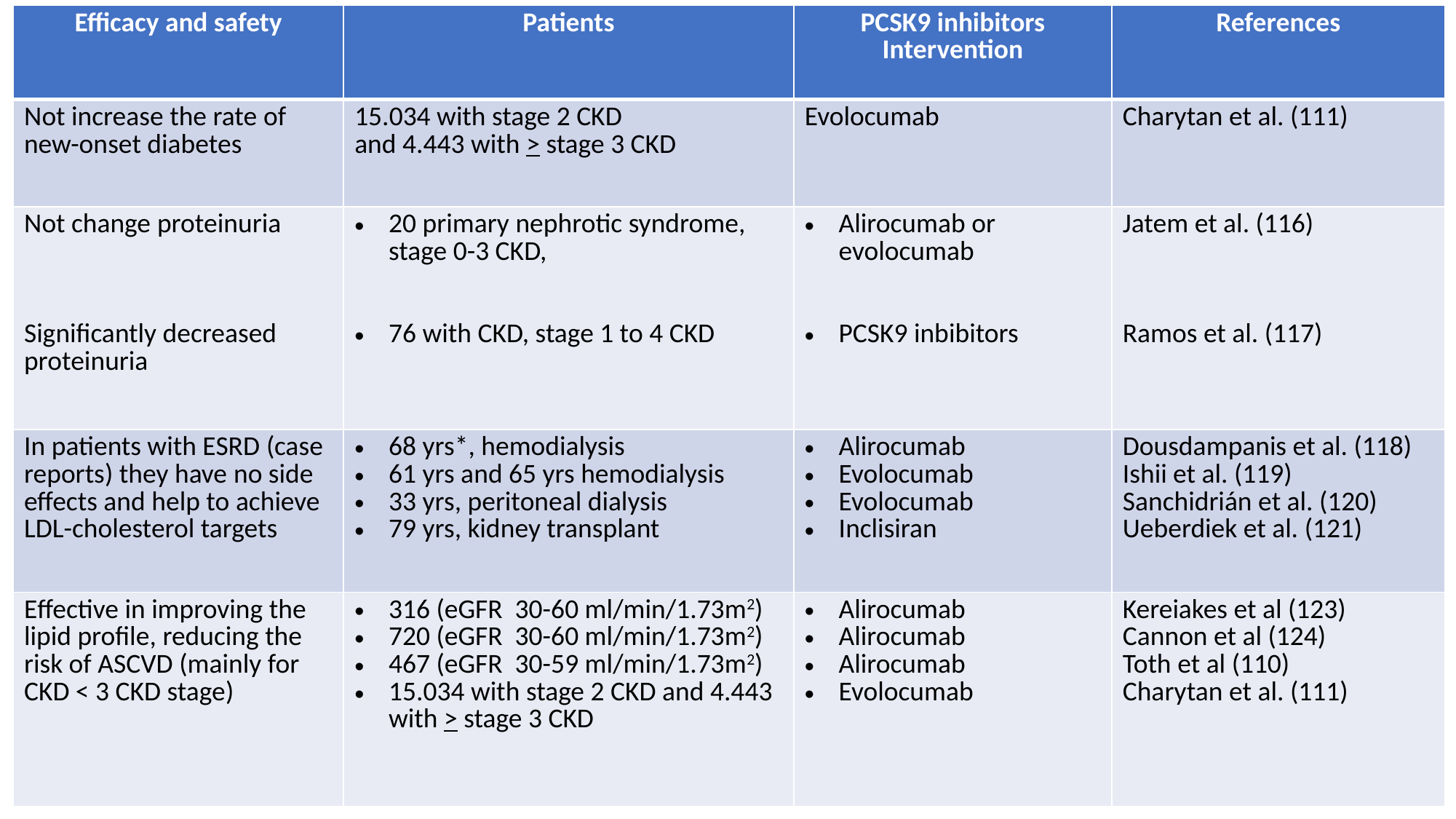

| Efficacy and safety | Patients | PCSK9 inhibitors Intervention | References |
| --- | --- | --- | --- |
| Not increase the rate of new-onset diabetes | 15.034 with stage 2 CKD and 4.443 with > stage 3 CKD | Evolocumab | Charytan et al. (111) |
| Not change proteinuria Significantly decreased proteinuria | 20 primary nephrotic syndrome, stage 0-3 CKD, 76 with CKD, stage 1 to 4 CKD | Alirocumab or evolocumab PCSK9 inbibitors | Jatem et al. (116) Ramos et al. (117) |
| In patients with ESRD (case reports) they have no side effects and help to achieve LDL-cholesterol targets | 68 yrs\*, hemodialysis 61 yrs and 65 yrs hemodialysis 33 yrs, peritoneal dialysis 79 yrs, kidney transplant | Alirocumab Evolocumab Evolocumab Inclisiran | Dousdampanis et al. (118) Ishii et al. (119) Sanchidrián et al. (120) Ueberdiek et al. (121) |
| Effective in improving the lipid profile, reducing the risk of ASCVD (mainly for CKD < 3 CKD stage) | 316 (eGFR 30-60 ml/min/1.73m2) 720 (eGFR 30-60 ml/min/1.73m2) 467 (eGFR 30-59 ml/min/1.73m2) 15.034 with stage 2 CKD and 4.443 with > stage 3 CKD | Alirocumab Alirocumab Alirocumab Evolocumab | Kereiakes et al (123) Cannon et al (124) Toth et al (110) Charytan et al. (111) |
