# Supplementary material for: Determinants of circulating PCSK9 levels and the efficacy of PCSK9 inhibitor therapies in chronic kidney disease: a systematic review
Source: Eur J Clin Pharmacol. 2026 Jan 17;82(2):37. doi: 10.1007/s00228-025-03965-w (PMC12812102; doi:10.1007/s00228-025-03965-w)
Supplement: Supplementary file 2 — Supplementary file2 (PPTX 57 KB) [file 228_2025_3965_MOESM2_ESM.pptx]

## Slide 1
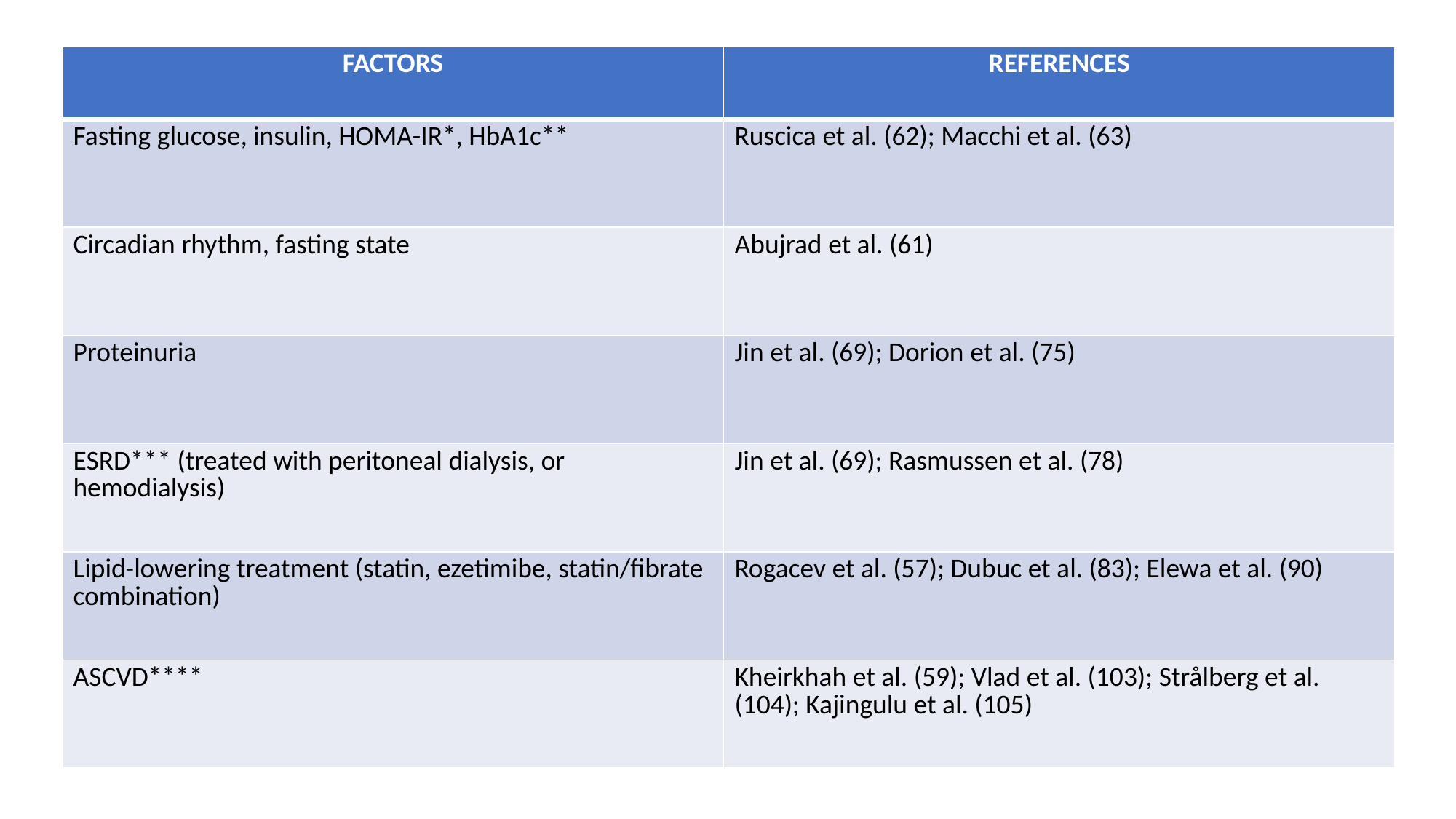

| FACTORS | REFERENCES |
| --- | --- |
| Fasting glucose, insulin, HOMA-IR\*, HbA1c\*\* | Ruscica et al. (62); Macchi et al. (63) |
| Circadian rhythm, fasting state | Abujrad et al. (61) |
| Proteinuria | Jin et al. (69); Dorion et al. (75) |
| ESRD\*\*\* (treated with peritoneal dialysis, or hemodialysis) | Jin et al. (69); Rasmussen et al. (78) |
| Lipid-lowering treatment (statin, ezetimibe, statin/fibrate combination) | Rogacev et al. (57); Dubuc et al. (83); Elewa et al. (90) |
| ASCVD\*\*\*\* | Kheirkhah et al. (59); Vlad et al. (103); Strålberg et al. (104); Kajingulu et al. (105) |

## Slide 2
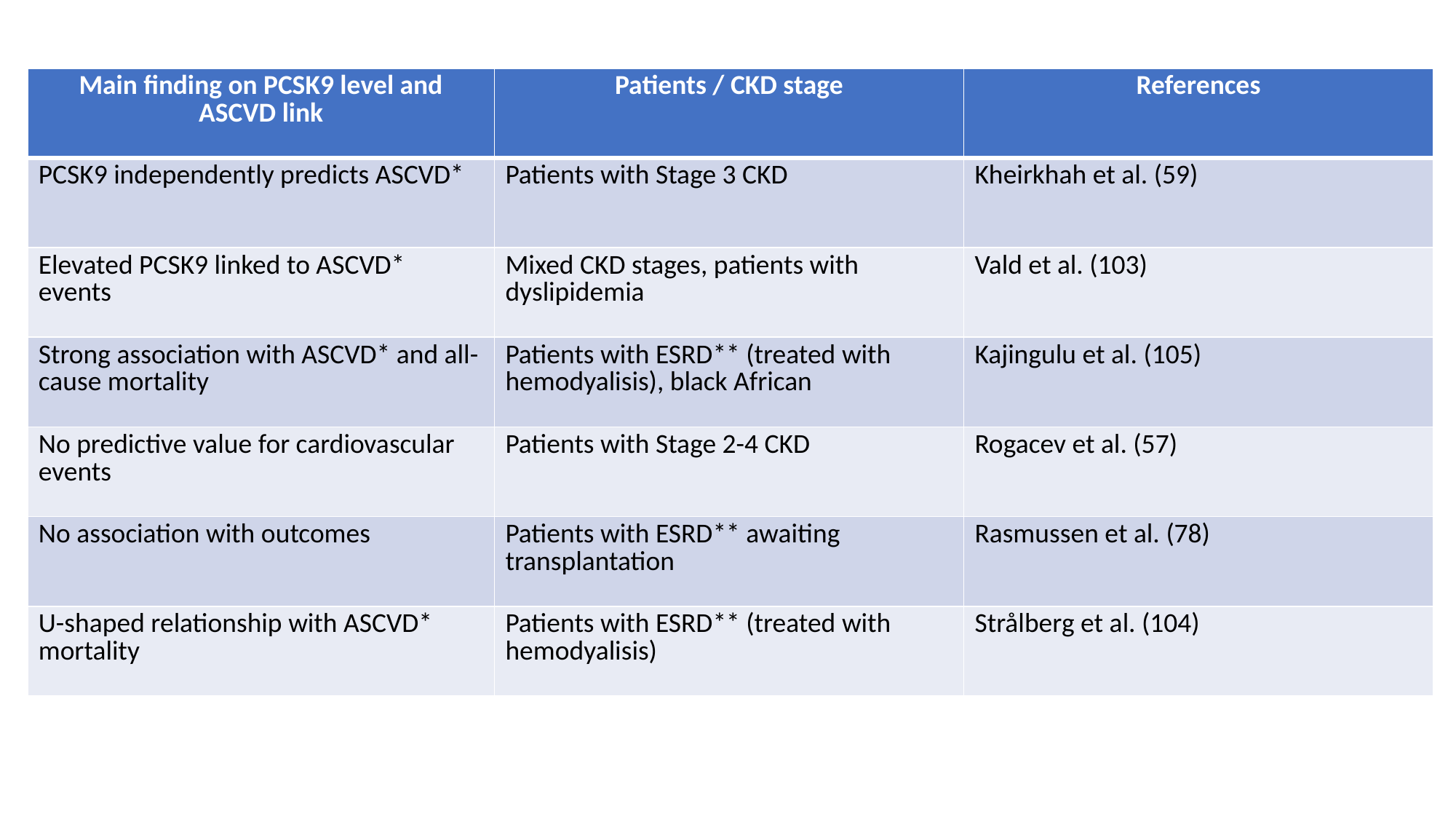

| Main finding on PCSK9 level and ASCVD link | Patients / CKD stage | References |
| --- | --- | --- |
| PCSK9 independently predicts ASCVD\* | Patients with Stage 3 CKD | Kheirkhah et al. (59) |
| Elevated PCSK9 linked to ASCVD\* events | Mixed CKD stages, patients with dyslipidemia | Vald et al. (103) |
| Strong association with ASCVD\* and all-cause mortality | Patients with ESRD\*\* (treated with hemodyalisis), black African | Kajingulu et al. (105) |
| No predictive value for cardiovascular events | Patients with Stage 2-4 CKD | Rogacev et al. (57) |
| No association with outcomes | Patients with ESRD\*\* awaiting transplantation | Rasmussen et al. (78) |
| U-shaped relationship with ASCVD\* mortality | Patients with ESRD\*\* (treated with hemodyalisis) | Strålberg et al. (104) |

## Slide 3
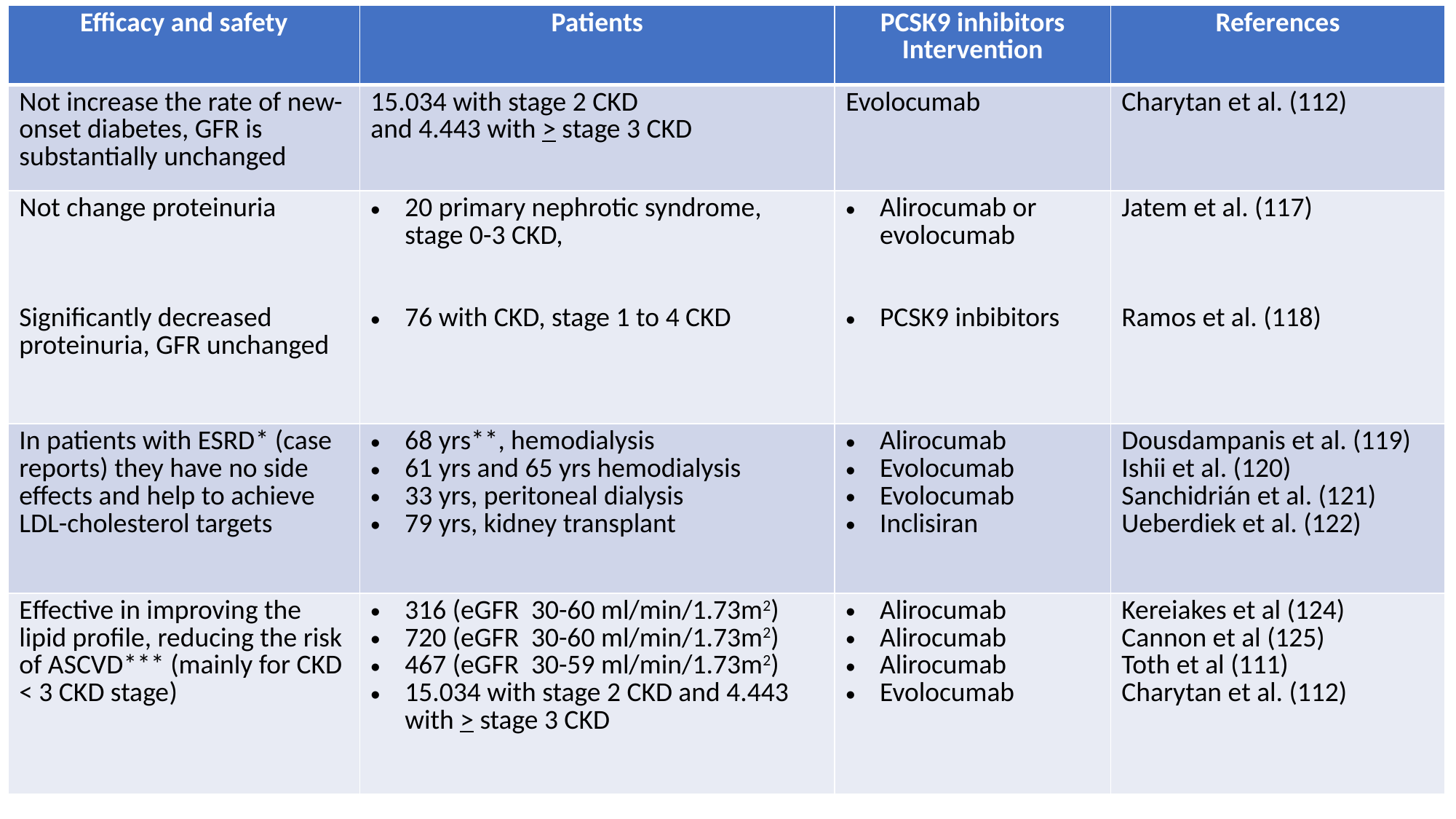

| Efficacy and safety | Patients | PCSK9 inhibitors Intervention | References |
| --- | --- | --- | --- |
| Not increase the rate of new-onset diabetes, GFR is substantially unchanged | 15.034 with stage 2 CKD and 4.443 with > stage 3 CKD | Evolocumab | Charytan et al. (112) |
| Not change proteinuria Significantly decreased proteinuria, GFR unchanged | 20 primary nephrotic syndrome, stage 0-3 CKD, 76 with CKD, stage 1 to 4 CKD | Alirocumab or evolocumab PCSK9 inbibitors | Jatem et al. (117) Ramos et al. (118) |
| In patients with ESRD\* (case reports) they have no side effects and help to achieve LDL-cholesterol targets | 68 yrs\*\*, hemodialysis 61 yrs and 65 yrs hemodialysis 33 yrs, peritoneal dialysis 79 yrs, kidney transplant | Alirocumab Evolocumab Evolocumab Inclisiran | Dousdampanis et al. (119) Ishii et al. (120) Sanchidrián et al. (121) Ueberdiek et al. (122) |
| Effective in improving the lipid profile, reducing the risk of ASCVD\*\*\* (mainly for CKD < 3 CKD stage) | 316 (eGFR 30-60 ml/min/1.73m2) 720 (eGFR 30-60 ml/min/1.73m2) 467 (eGFR 30-59 ml/min/1.73m2) 15.034 with stage 2 CKD and 4.443 with > stage 3 CKD | Alirocumab Alirocumab Alirocumab Evolocumab | Kereiakes et al (124) Cannon et al (125) Toth et al (111) Charytan et al. (112) |
